# External validation of a multi-biomarker-based score for predicting risk of cardiovascular disease in patients with rheumatoid arthritis

**DOI:** 10.1371/journal.pone.0296459

**Published:** 2024-05-06

**Authors:** Eric H. Sasso, Brent Mabey, Darl D. Flake, Elena Hitraya, Cheryl L. Chin, Rotem Ben-Shachar, Alexander Gutin, Jerry S. Lanchbury, Jeffrey R. Curtis

**Affiliations:** 1 Medical and Scientific Affairs, Crescendo Bioscience, South San Francisco, CA, United States of America; 2 Myriad Genetics Laboratories, Myriad, Salt Lake City, UT, United States of America; 3 Division of Clinical Immunology and Rheumatology, University of Alabama at Birmingham, Birmingham, AL, United States of America; University of Toronto, CANADA

## Abstract

**Background:**

A multi-biomarker disease activity (MBDA)-based cardiovascular disease (CVD) risk score was developed and internally validated in a Medicare cohort to predict 3-year risk for myocardial infarction (MI), stroke or CVD death in patients with rheumatoid arthritis (RA). It combines the MBDA score, leptin, MMP-3, TNF-R1, age and four clinical variables. We are now externally validating it in a younger RA cohort.

**Methods:**

Claims data from a private aggregator were linked to MBDA test data to create a cohort of RA patients ≥18 years old. A univariable Cox proportional hazards regression model was fit using the MBDA-based CVD risk score as sole predictor of time-to-a-CVD event (hospitalized MI or stroke). Hazard ratio (HR) estimate was determined for all patients and for clinically relevant subgroups. A multivariable Cox model evaluated whether the MBDA-based CVD risk score adds predictive information to clinical data.

**Results:**

49,028 RA patients (340 CVD events) were studied. Mean age was 52.3 years; 18.3% were male. HR for predicting 3-year risk of a CVD event by the MBDA-based CVD risk score in the full cohort was 3.99 (95% CI: 3.51–4.49, p = 5.0×10^−95^). HR were also significant for subgroups based on age, comorbidities, disease activity, and drug use. In a multivariable model, the MBDA-based CVD risk score added significant information to hypertension, diabetes, tobacco use, history of CVD, age, sex and CRP (HR = 2.27, p = 1.7×10^−7^).

**Conclusion:**

The MBDA-based CVD risk score has been externally validated in an RA cohort that is younger than and independent of the Medicare cohort that was used for development and internal validation.

## Background

Cardiovascular disease (CVD) accounts for 30−40% of deaths among patients with rheumatoid arthritis (RA) and is their leading cause of mortality [1]. CVD incidence among RA patients is 50% greater than in the general population [2]. Cardiovascular risk calculators developed for the general population tend to underestimate CVD risk for RA patients [3–5], leading the European League Against Rheumatism to recommend that CVD risk estimates from conventional calculators be multiplied by 1.5 for RA patients [3]. This adjustment does not account for the effect of RA inflammation, which varies between patients and can meaningfully increase the risk for atherosclerosis and CVD events.

To improve CVD risk prediction for RA patients, a CVD risk score based on clinical information and the multi-biomarker disease activity (MBDA) blood test has been developed [6]. The MBDA test provides a validated score for assessing and monitoring RA disease activity, based on measurements of 12 serum biomarkers [7]. In 2019, the American College of Rheumatology disease activity measures working group concluded that the MBDA score was one of the measures of RA disease activity that met the minimum standard for regular use [8]. The MBDA-based CVD risk score combines the MBDA score with three of its biomarkers (leptin, MMP-3 and TNR-R1), age, and four traditional clinical CVD risk factors (diabetes, hypertension, tobacco use and history of a high-risk cardiovascular condition) to predict the 3-year risk for myocardial infarction (MI), stroke or CVD death in patients with RA [6].

The MBDA-based CVD risk score was developed and internally validated in a cohort of 30,751 RA patients in the US that was created by linking Medicare administrative data with MBDA test results [6]. Comorbidity rates were high in this cohort, as 77% of the patients were at least 65 years old. The MBDA-based CVD risk score was also internally validated in subgroups of Medicare patients, including those who were <65 years old, lacked comorbidities or were already receiving statins.

Many rheumatologists do not routinely assess RA disease activity quantitatively or formally evaluate non-rheumatologic CVD risk factors. These barriers may be overcome if, as a routine part of MBDA testing, the MBDA-based CVD risk score were to be generated automatically by the MBDA laboratory after it measures the MBDA biomarker concentrations and produces an MBDA score. Rheumatologists may then find the MBDA-based CVD risk score easier to use than conventional CVD risk scores because it would not require them to assess cholesterol or other clinical measures or perform any calculations.

In view of this potential opportunity to streamline CVD risk assessment in RA by using the MBDA-based CVD risk score, we conducted the present analysis of commercial claims data from private payors in the US to provide an independent, external validation of the MBDA-based CVD risk score in a younger, non-Medicare cohort.

## Methods

### Data source

A retrospective cohort was created by using all available fee-for-service data from a population of RA patients who had received MBDA testing as part of routine care and for whom records for medical and pharmaceutical claims were available. Medical and pharmaceutical claims data came from the Integrated Dataverse^®^ of Symphony Health (Phoenix, AZ, USA), which longitudinally assembles data from medical, hospital and prescription claims, point-of-sale prescription data, non-retail invoice data and demographic data (https://symphonyhealth.prahs.com/what-we-do/view-health-data). MBDA test results came from a commercial database (Vectra^®^, Crescendo Bioscience, Inc., South San Francisco, CA, USA; subsequently owned by Myriad Genetics Laboratories, Salt Lake City, UT, USA), followed by LabCorp (Burlington, NC, USA). For this study, MBDA data from September 30, 2010 to April 30, 2018 were sent to Symphony Health, where matched medical and pharmaceutical claims data from January 1, 2011 to December 31, 2017 were linked to MBDA test data and sent to the authors as a deidentified dataset. The data in the de-identified, linked dataset that was analyzed here had been collected independently of and prior to this study, by individuals other than the investigators, from individuals who were not identifiable by the investigators. Based on 45 CFR 46.102(e) [1–6], the study was exempted from human subjects review.

### Eligibility criteria

Patients were considered for inclusion if they had received ≥1 MBDA test and had any medical and pharmaceutical claims data available from ≥365 days before the date of the qualifying MBDA test (see below). For a patient to be included, the linked database had to: 1) contain a diagnosis of RA by a rheumatologist, using International Statistical Classification of Diseases and Related Health Problems (ICD) diagnosis codes, 9^th^ or 10^th^ revision (ICD9 714.0; ICD10 M05.*, M06.*, excluding M06.4 and M06.1, with * representing any number of digits or characters), before the qualifying MBDA test date and 2) indicate receipt of an RA-specific treatment (TNF-inhibitor, abatacept, rituximab, anti-IL-6R, Janus kinase inhibitor, conventional synthetic disease-modifying anti-rheumatic drug [csDMARD] including methotrexate, sulfasalazine, leflunomide and hydroxychloroquine) on or before the MBDA test date.

A patient was excluded if they had listed Medicare insurance on their MBDA test requisition form, to avoid overlap with the cohort used to develop the MBDA-based CVD risk score; or if their MBDA tests were all excluded, based on any of the following criteria: 1) hospitalization during the 14 days before the test date, 2) anti-IL-6R therapy received during the 90 days on or before the test date [9], 3) malignant neoplasm (except non-melanoma skin cancer) ≤365 days before the test date, or 4) CVD event (defined below) before the test date. For patients with multiple MBDA tests, the earliest qualifying test was used.

### CVD outcome

The outcome for validating the MBDA-based CVD risk test was a hospitalized CVD event, defined as a hospital claim for MI or stroke, based on ICD-9 or ICD-10 codes (S1 Table). CVD death information was unavailable in this data source and was not part of the composite outcome. The endpoint used to analyze the composite outcome was time from MBDA testing to first CVD event. Follow-up for each patient spanned from the qualifying MBDA test date to the earliest of: 1) first CVD event, 2) malignant neoplasm (except non-melanoma skin cancer), 3) three years after MBDA testing, or 4) date of the last medical or pharmaceutical claim.

### MBDA-based CVD risk score

#### Clinical variables

Clinical variables in the MBDA-based CVD risk score are age at the time of qualifying MBDA test, obtained from the MBDA database; and diagnoses of diabetes, hypertension, tobacco use (past or present) and history of CVD other than MI or stroke, based on nine CV diagnoses established previously [6]. Each diagnosis was considered present or absent based on diagnostic codes in medical and hospital claims on or before the MBDA test date (S1 Table).

#### Molecular variables

Molecular variables in the MBDA-based CVD risk score are: leptin, MMP-3, TNF-R1 and the MBDA score. The MBDA score measures RA disease activity with 12 protein biomarkers (EGF, VEGF-A, MMP-1, MMP-3, TNF-RI, VCAM-1, SAA, YKL-40, CRP, IL-6, leptin and resistin), using a validated algorithm to give an integer score on a scale of 1 to 100 with categories of Low (<30), Moderate (30–44) and High (≥45) [7]. All MBDA tests used in this study were ordered as part of routine patient care, with testing performed in a Clinical Laboratory Improvements Amendment-certified commercial laboratory in South San Francisco, CA, USA (Crescendo Bioscience). Values for leptin, MMP-3, TNF-R1 and CRP came from MBDA tests.

Since 2017, the original MBDA score has been routinely adjusted for age, sex, and adiposity [10]. The adjusted MBDA score has the same scale, categories and minimally important difference (8 units) as the original MBDA score [10]. Original MBDA scores were converted to adjusted scores for this study. Hereafter, “MBDA score” means the adjusted MBDA score.

#### The MBDA-based CVD risk score algorithm

MBDAbasedCVDriskscore=0.031441×age+0.273186×diabetes+0.269370×hypertension+0.269117×smoking+0.337822×CVDHistory−0.171106×ln(Leptin)+0.145355×ln(MMP3)+0.572441×ln(TNFRI)+1.607582×tanh(MBDA/33.08073).

Age is in years, leptin, MMP-3, and TNF-RI are in ng/mL, ln means natural logarithm and tanh represents hyperbolic tangent transformation [6]. Having leptin, MMP-3 and TNF-R1 as separate variables in the MBDA-based CVD risk score and as components within the MBDA score is non-redundant because the algorithm for the MBDA score is a weighted, non-linear combination of its component biomarkers, which were originally neither selected nor weighted for CVD risk prediction [6, 7, 10]. A separate formula that converts the MBDA-based CVD risk score into predicted 3-year risk for a CVD event as a percentage value [6] was not used here.

### Statistical analysis

#### Objectives

The pre-defined primary objective was to validate the MBDA-based CVD risk score as a predictor of CVD events. Secondary objectives included validating the score in patients <65 years old and evaluating whether the MBDA-based CVD risk score adds prognostic information to clinical variables.

Symphony Health estimates that the Integrated Dataverse includes approximately 60% of medical claims, 60% of mail order pharmaceutical claims and 81% of retail pharmaceutical claims (data on file). Assuming that the Integrated Dataverse misses data randomly, the relationships between CVD event rates and molecular, clinical and other variables should be more reliable than absolute event rates. The present analyses thus focused on predicting relative risk of a CVD event rather than absolute risk.

#### Validation

A univariable Cox proportional hazards regression model was fit by using the MBDA-based CVD risk score as the sole predictor of time to a CVD event. The hazard ratio (HR) estimate was determined, with p-value and 95% confidence interval based on the partial likelihood ratio test (LRT). This methodology was also used in patients <65 years old and ≥40 years old and in exploratory analyses for other subgroups based on molecular, clinical, therapeutic and demographic features. To determine if HR differed between complementary subgroups, p-value was determined using the partial LRT, with Bonferroni correction for multiple comparisons.

A multivariable Cox model was fit using the following covariates to predict time to CVD event: MBDA-based CVD risk score, age, sex, CRP (natural log transformed), diabetes yes/no (Y/N), hypertension Y/N, history of other CVD Y/N and tobacco use Y/N. HR and LRT-based p-value were reported for the MBDA-based CVD risk score after accounting for all other variables in the multivariable model.

Analyses were performed using R (version 3.5.1) and the survival (version 3.1–8) package [11, 12].

## Results

A total of 49,028 RA patients with 340 CVD events (165 MIs and 175 strokes) met eligibility criteria (S2 Table). Mean age was 52.3 years, 18.3% of patients were male (Table 1). Hypertension (39.1%), hyperlipidemia (38.2%) and diabetes (16.3%) were the most frequent CVD-related co-morbidities (Table 1). Use of csDMARDs and biologics was similar among patients with and without a CVD event.

**Table 1:** Patient characteristics at baseline*.

Characteristic	Complete cohort	Patients with CVD event	Patients with no CVD event
	*N = 49*,*028*	*N = 340*	*N = 48*,*688*
**Age, mean (SD)**	52.3 (11.2)	61.6 (11.6)	52.2 (11.2)
**Age group, %**			
<40 years	6,720 (13.7%)	11 (3.2%)	6,709 (13.8%)
40–64 years	37,805 (77.1%)	207 (60.9%)	37,598 (77.2%)
65–74 years	3466 (7.1%)	66 (19.4%)	3400 (7.0%)
≥75 years	1037 (2.1%)	56 (16.5%)	981 (2.0%)
**Male, N (%)**	8972 (18.3%)	105 (30.9%)	8867 (18.2%)
**Comorbidities, N (%)**			
Diabetes	8002 (16.3%)	133 (39.1%)	7869 (16.2%)
History of CVD	6730 (13.7%)	151 (44.4%)	6579 (13.5%)
Hyperlipidemia	18,731(38.2%)	209 (61.5%)	18,522 (38.0%)
Hypertension	19,189 (39.1%)	247 (72.6%)	18,942 (38.9%)
Tobacco use (past or current)	7517 (15.3%)	104 (30.6%)	7413 (15.2%)
**Medications, N (%)**			
ACEI	6099 (12.4%)	87 (25.6%)	6012 (12.3%)
ARB	4478 (9.1%)	58 (17.1%)	4420 (9.1%)
Beta-blockers	6397 (13.0%)	97 (28.5%)	6300 (12.9%)
Statins	5815 (11.9%)	85 (25.0%)	5730 (11.8%)
**RA medications, N (%)**			
Methotrexate	21,798 (44.4%)	153 (45%)	21,645 (44.4%)
Other csDMARDs	16,436 (33.5%)	120 (35.3%)	16,316 (33.5%)
TNFi biologics	11,370 (23.2%)	73 (21.5%)	11,297 (23.2%)
Non-TNFi biologics	3374 (6.9%)	21 (6.2%)	3353 (6.9%)
Abatacept	1841 (3.8%)	10 (2.9%)	1831 (3.8%)
Rituximab	402 (0.8%)	4 (1.2%)	398 (0.8%)
Tocilizumab	179 (0.4%)	3 (0.9%)	176 (0.4%)
Tofacitinib	1038 (2.1%)	5 (1.5%)	1033 (2.1%)
Oral Glucocorticoids	24,154 (49.3%)	196 (57.6%)	23,958 (49.2%)
NSAIDs	13,327 (27.2%)	104 (30.6%)	13,223 (27.2%)
**Biomarkers** † **, median (IQR)**			
Leptin, (ng/ml)	24.3 (10.6–47.1)	23.7 (9.1–47.8)	24.3 (10.6–47.1)
MMP-3, (ng/ml)	21.1 (14.2–36.3)	28.7 (18.7–57.1)	21.0 (14.2–36.1)
TNF-R1, (ng/ml)	1.4 (1.1–1.7)	1.8 (1.4–2.7)	1.4 (1.1–1.7)
CRP (mg/L)	4.1 (1.4–11.6)	6.3 (2.1–16.7)	4.1 (1.4–11.5)
**MBDA score, median (IQR)**	40 (31–48)	44 (34–52)	40 (31–48)

*Based on diagnostic codes and administration and fill information in the baseline period (see Methods).

†Biomarker concentrations are from the MBDA test. Leptin, MMP-3 and TNF-R1 values are natural log transformed.

CVD event is hospitalized myocardial infarction or stroke in the three years from the date of the baseline MBDA score.

ACEI, angiotensin converting enzyme inhibitor; ARB, angiotensin receptor blocker; csDMARD, conventional synthetic disease modifying antirheumatic drug; CVD, cardiovascular disease; IQR, interquartile range; MBDA, MMP-3, matrix metalloproteinase protein 3; multi-biomarker disease activity (adjusted); NSAID, non-steroidal anti-inflammatory drug; SD, standard deviation; TNFi, tumor necrosis factor inhibitor; TNF-R1, TNF receptor 1.

Total follow-up time was 93,514 patient-years (PY), with median (interquartile range) follow-up duration of 24.4 (12.2–36.0) months. The overall observed CVD event rate was 3.6 events/1000 PY.

### Validation of the MBDA-based CVD risk score

**Fig 1:**
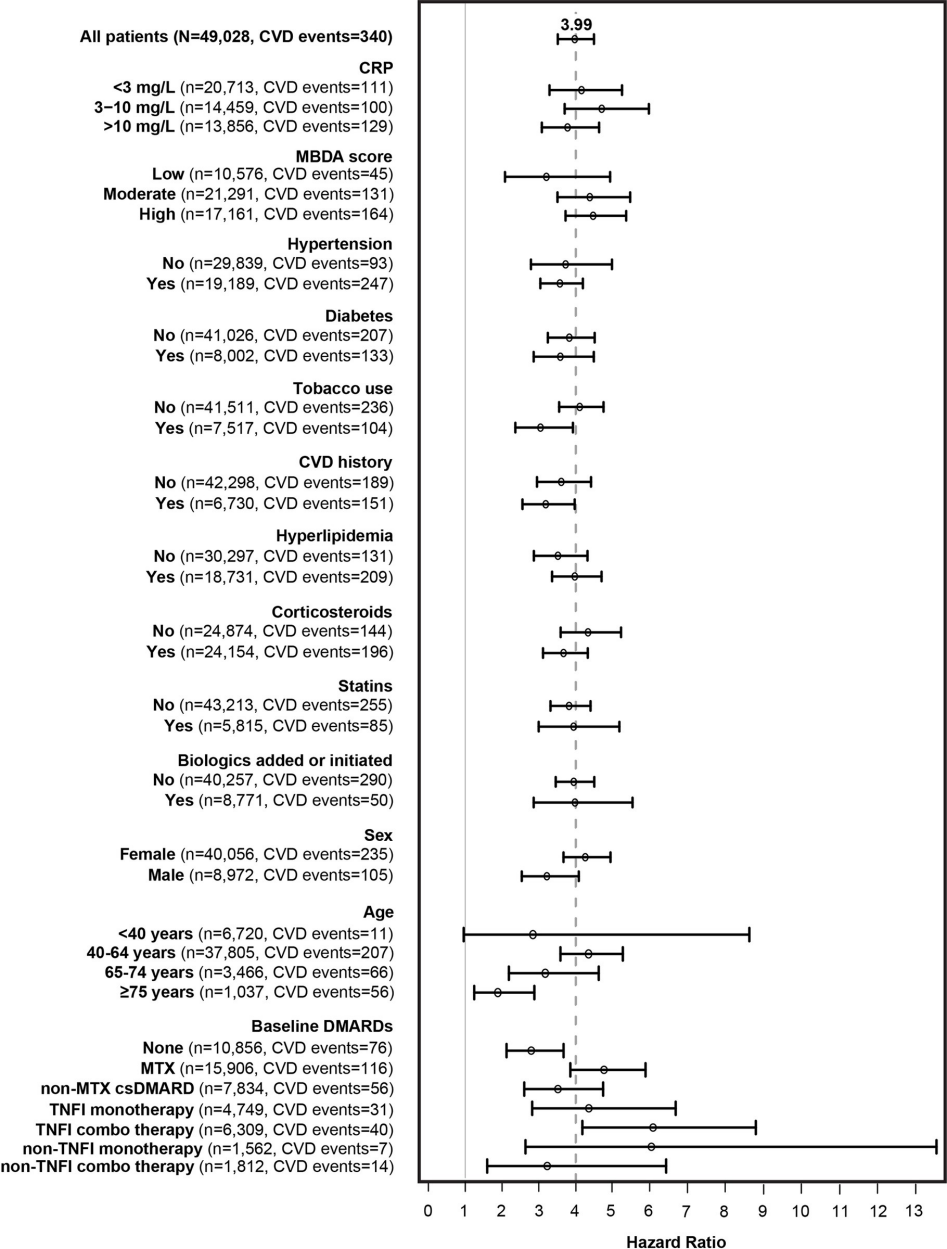
Validation of MBDA-based CVD risk score for predicting CVD risk in subgroups. Results show hazard ratios (HRs) with 95% confidence intervals. Subgroupings are based on information from baseline period, except Biologic initiation or change, which is based on treatment changes during follow-up. Vertical lines indicate HRs of 1.0 (solid line) and 3.99 (dashed line). All HRs are statistically significantly >1.0, except for the <40-year-old subgroup. Comparisons between complementary subgroups all have p >0.05 except for Hypertension (p = 0.046), Sex (p = 0.046), Baseline drug use (p = 0.012) and Age (p = 0.005), which are each non-significant after Bonferroni correction for 13 comparisons (i.e., p >0.0038 [0.05/13]). For baseline RA drugs, the Methotrexate (MTX) category includes combinations with conventional synthetic DMARDs (csDMARDs); the non-MTX csDMARD category excludes combinations with MTX, and the non-TNF inhibitor (TNFi) monotherapy (mono); and combination therapy (combo) categories include Janus kinase inhibitors.

The MBDA-based CVD risk score was a significant predictor of 3-year CVD risk in univariable analysis, with HR of 3.97 (95% CI: 3.51–4.49, p = 5.0×10^−95^) (Fig 1), indicating that for every one-unit increase in the risk score, CVD risk was approximately four times as large. For the patients <65 years old (N = 44,525 with 218 CVD events), HR was 4.23 (95% CI: 3.51–5.09, p = 6.4×10^−48^). For patients ≥40 years old (N = 42,308 with 329 CVD events), HR was 4.00 (95% CI: 3.52–4.55, p = 1.1×10^−85^). For patients 65 to 74 years old, HR was 3.17 (Fig 1), which resembles the 2.89 value observed previously in the overall Medicare cohort, with a mean age of 69 years [6].

HRs for the MBDA-based CVD risk score were statistically significantly and markedly >1.0 for nearly all subgroups of interest, with most HRs being between 3.0 and 4.5 (Fig 1). Results for subgroups with small event numbers, such as patients <40 years old and patients receiving non-TNFi biologics, should be interpreted with caution. HRs were generally similar between complementary subgroups, such as patients with and without diabetes, with none being statistically significantly different following correction for multiple testing (Fig 1). In these comparisons of HRs of complementary subgroups, a higher HR does not mean greater CVD risk. Rather, it means that, for any increase in MBDA-based CVD risk score, CVD risk increases more for patients in the subgroup with the greater HR than in the complementary subgroup.

When a multivariable model was fit with the MBDA-based CVD risk score and seven demographic or clinical CVD risk factors (age, sex, CRP, diabetes, hypertension, history of other CVD, and tobacco use), the MBDA-based CVD risk score added significant, independent predictive information to the model, with an HR of 2.27 (95% CI: 1.68–3.06, p = 1.7×10^−7^) (Table 2). Within this model, leptin, MMP-3, TNF-R1 and the MBDA score are unique to the MBDA-based CVD risk score. Thus, this result indicates that biomarker information in the MBDA-based CVD risk score contributes prognostic information that was not captured by the ensemble of conventional CVD risk factors in the model.

**Table 2:** Univariable and multivariable analyses of the MBDA-based CVD risk score and conventional CVD risk factors as predictors of risk for a CVD event.

	Univariable Analyses		Multivariable Model	
Variable	HR (95% CI)	P-value	HR (95% CI)	P-value
Age	1.08 (1.07, 1.09)	6.4×10^−53^	1.02 (1.01, 1.04)	0.007
Sex (male)	2.02 (1.60, 2.54)	1.3×10^−8^	1.17 (0.92, 1.49)	0.204
Ln (CRP)	1.19 (1.11, 1.28)	8.2×10^−7^	0.98 (0.90, 1.06)	0.580
Hypertension	4.01 (3.16, 5.09)	1.6×10^−34^	1.37 (1.04, 1.81)	0.025
Smoking	2.77 (2.20, 3.49)	1.3×10^−15^	1.69 (1.32, 2.18)	6.7×10^−5^
Diabetes	3.37 (2.71, 4.19)	1.7×10^−24^	1.41 (1.10, 1.80)	0.007
CVD History	5.22 (4.22, 6.47)	1.1×10^−44^	1.56 (1.19, 2.04)	0.001
MBDA-based CVD risk score	3.99 (3.51–4.49)	4.4×10^−95^	2.27 (1.68, 3.06)	1.7×10^−7^

Cardiovascular disease (CVD) event is defined as hospitalized event or stroke in the three years following the baseline MBDA score. CRP, C-reactive protein; HR, hazard ratio; ln, natural logarithm.

## Discussion

An MBDA-based CVD risk score that combines molecular, clinical and demographic features to provide a personalized assessment of CVD risk for patients with RA was previously developed and internally validated in a predominantly elderly cohort of Medicare patients. Only 23% of those patients were <65 years old, of whom most had disability [6]. We have now externally validated the MBDA-based CVD risk score in an independent RA cohort that was younger and had lower rates of comorbidities than the Medicare cohort. We also validated the MBDA-based CVD risk score in several subgroups, based on comorbidities, drug use and level of inflammatory disease activity. In addition, we showed that the risk score improved the predictive ability of a simpler, clinically-based model. This study thus supports the MBDA-based CVD risk score as a feasible tool that objectively evaluates inflammatory disease activity to assess CVD risk in a heterogeneous RA patient population.

Previously, the MBDA-based CVD risk score was developed and internally validated for RA patients ≥40 years old, to align with ACC/AHA guidelines [6, 13]. The present cohort included RA patients ≥18 years old to validate the MBDA-based CVD risk score in the age range of patients who may receive the MBDA test, which is required to calculate the MBDA-based CVD risk score. For completeness, the MBDA-based CVD risk score was also validated in patients ≥40 years old and patients <65 years old. These analyses thus expand the overall age range in which the MBDA-based CVD risk score has been studied while also validating it in the same age group that was used for internal validation in the Medicare cohort.

The present cohort was created by linking the MBDA database to clinical data from a large administrative database, the Integrated Dataverse of Symphony Health. A limitation of this cohort is that neither non-RA patients nor RA patients who had not received MBDA testing could be studied because MBDA testing is only for RA patients and MBDA test data are required for the MBDA-based CVD risk score. The Symphony database does not capture all claims for all patients, and it does not contain information on cardiovascular death, which is part of the composite outcome the MBDA-based CVD risk score was designed to predict. The lack of CVD death data probably did not meaningfully affect the accuracy of CVD event rates in this study because CVD death comprised only 6.9% of CVD events in the Medicare database used previously, and it may comprise a smaller portion in the younger cohort used here. We do not expect the missingness of data in Symphony to be related to MBDA-based CVD risk scores. Thus, while the absolute incidence rate of CVD events in our cohort, ~4/1000 PY, is lower than might be expected [14], co-morbidity rates resembled those seen elsewhere [15] and the HR values in Fig 1, being assessments of relative risk, should be unbiased with respect to the MBDA-based CVD risk score.

A limitation of this study is that the MBDA-based CVD risk score could not be compared to conventional CVD risk calculators, such as the Framingham Risk Score or the pooled cohort risk equation of the American College of Cardiology and American Heart Association, because the clinical measurements they require are not available in the Symphony claims database. Instead, we created a multivariable model that combined age, sex, CRP and four clinical diagnosis variables − each weighted optimally for CVD risk prediction in the current cohort − with the MBDA-based CVD risk score included in the model as an additional variable. The MBDA-based CVD risk score added significant information to this model, indicating that biomarker-based information in the MBDA-based CVD risk score, which represents RA inflammation, contributed prognostic information that was not captured by the seven clinically-based variables, which included CRP.

Other models have been developed to predict risk for CVD events in RA patients. The Expanded Cardiovascular Risk Prediction Score (ERS-RA), which predicts 10-year CVD risk and has been externally validated [16, 17], combines six demographic/CVD-related factors, many of which are also in the MBDA-based risk prediction score, with 4 RA-related variables, including the Clinical Disease Activity Index (CDAI) to assess disease activity and the Health Assessment Questionnaire (HAQ), for disability. The Trans-Atlantic Cardiovascular Risk Consortium for Rheumatoid Arthritis (ATACC-RA) developed two models predicting 10-year CVD risk that require serum lipid measurements and account for RA-related effects with the 28-joint Disease Activity Score with erythrocyte sedimentation rate (DAS28-ESR) or the HAQ, respectively [18]. More recently, another group developed and externally validated models for 2-year risk for MI and for stroke in RA patients who are initiating first-line methotrexate monotherapy, using 64 and 90 predictor variables, respectively, but no clinical measures [19]. The MBDA-based CVD risk score uses nine variables and requires no RA-related clinical measures and no blood test results, except from the MBDA test. If the MBDA-based CVD risk score were to be automatically calculated by the MBDA laboratory at the time of MBDA testing, it may provide rheumatologists a practical way to assess CVD risk for their RA patients.

## Conclusions

In conclusion, we have externally validated the MBDA-based CVD risk score in an independent cohort that is younger than the Medicare cohort that was used for test development. The score was shown to discriminate 3-year CVD risk among patients >18 years old, ≥40 years old or <65 years old, and in various clinical subgroups. These results support the feasibility and potential utility of using biomarker measurements to personalize CVD risk assessment according to the level of RA inflammation.

## Supporting information

S1 Table Diagnosis codes used to identify cardiovascular events and CVD risk factors in the linked database. * Represents any number of digits or characters. †See reference [6] (Curtis et al, 2020). CVD, cardiovascular disease; ICD, International Statistical Classification of Diseases and Related Health Problems, MI, myocardial infarction.
(DOCX)

S2 Table Cohort derivation. * Represents any number of digits or characters. †See reference [6] (Curtis et al, 2020). CVD, cardiovascular disease; ICD, International Statistical Classification of Diseases and Related Health Problems, MI, myocardial infarction.
(DOCX)

## List of abbreviations

ACEI: angiotensin converting enzyme inhibitor
ARB: angiotensin receptor blocker
csDMARD: conventional synthetic disease modifying antirheumatic drug
CVD: cardiovascular disease
CRP: C-reactive protein
HR: hazard ratio
IQR: interquartile range
ln: natural logarithm
MBDA: multi-biomarker disease activity (adjusted
MMP-3: matrix metalloproteinase 3
NSAID: non-steroidal anti-inflammatory drug
PY: patient-years
RA: rheumatoid arthritis
SD: standard deviation
TNFi: tumor necrosis factor inhibitor
TNF-R1: tumor necrosis factor receptor 1
