# Supplementary material for: External validation of a multi-biomarker-based score for predicting risk of cardiovascular disease in patients with rheumatoid arthritis
Source: PLoS One. 2024 May 6;19(5):e0296459. doi: 10.1371/journal.pone.0296459 (PMC11073667; doi:10.1371/journal.pone.0296459)
Supplement: S1 Table — * Represents any number of digits or characters. †See reference [6] (Curtis et al, 2020). CVD, cardiovascular disease; ICD, International Statistical Classification of Diseases and Related Health Problems, MI, myocardial infarction. (DOCX) [file pone.0296459.s001.docx]

# SUPPLEMENTARY MATERIAL

# Supplemental Table 1. Diagnosis codes used to identify cardiovascular events and CVD risk factors in the linked database.

| **Diagnosis** | **ICD-9 Codes** | **ICD-10 Codes** |
| --- | --- | --- |
| **Myocardial infarction** | 410.*1 | I21.* |
| **Stroke** | 430.*, 431.*, 433.*1, 434.*1, 436.* | I60.*, I61.*, I63.*, I67.89 |
| **Diabetes** | 250.*, 357.2*, 362.0*, 366.41 | E10.*, E11.*, E08.*, E09.* |
| **Hypertension** | 401.* | I10.* |
| **History of CVD other than MI or stoke†** | 427.31, 441.*, 440.*, 444.81, 444.2*, 411.*, 412.*, 413.*, 414.*, 428.*, 432.*, 435.* | I48.91, I71.*, I70.2*, I70.499, I74.*, I24.*, I20.*, I25.*, I50.*, I62.*, G45.0, G45.1, G45.8, G45.9, I67.848 |
| **Tobacco use** | 305.1*, V15.82 | F17.2*, Z87.891 |

* Represents any number of digits or characters. †See reference 7 (Curtis et al, 2020).

CVD, cardiovascular disease; ICD, International Statistical Classification of Diseases and Related Health Problems, MI, myocardial infarction.
