# Supplementary material for: External validation of a multi-biomarker-based score for predicting risk of cardiovascular disease in patients with rheumatoid arthritis
Source: PLoS One. 2024 May 6;19(5):e0296459. doi: 10.1371/journal.pone.0296459 (PMC11073667; doi:10.1371/journal.pone.0296459)
Supplement: S2 Table — * Represents any number of digits or characters. †See reference [6] (Curtis et al, 2020). CVD, cardiovascular disease; ICD, International Statistical Classification of Diseases and Related Health Problems, MI, myocardial infarction. (DOCX) [file pone.0296459.s002.docx]

# SUPPLEMENTARY MATERIAL

**Supplemental Table 2. Cohort derivation.**

| **Selection step** | **Number of patients** | **Number of observations** |
| --- | --- | --- |
| Vectra data (2010 to 4/30/18) sent to Symphony | N/A | N/A |
| Symphony data (1/1/2011 to 12/31/17) linked to Vectra data | 358,640 | 721,494 |
| Require at least 1 medical claim and 1 pharmaceutical claim at least 365 days prior to Vectra test | 303,533 | 597,562 |
| Require a diagnosis of RA prior to Vectra test | 112,238 | 238,948 |
| Require RA-specific treatment prior to Vectra test | 103,194 | 221,192 |
| Exclude subjects with recent hospitalization, anti-IL-6R treatment, prior MI or stroke, malignancy prior to Vectra test | 97,443 | 203,614 |
| Exclude patients with Medicare insurance | 55,456 | 105,983 |
| Take first Vectra test | 55,456 | 55,456 |
| Exclude patients with no follow-up after Vectra test | 49,028 | 49,028 |

* Represents any number of digits or characters. †See reference 7 (Curtis et al, 2020).

CVD, cardiovascular disease; ICD, International Statistical Classification of Diseases and Related Health Problems, MI, myocardial infarction.
